# Gas Beyond the Lumen: Gastric Pneumatosis With Portal Venous Gas Following Blunt Abdominal Trauma

**DOI:** 10.7759/cureus.106116

**Published:** 2026-03-30

**Authors:** Harsh V Baranwal, Muskan Dugar, Ronit Biswas, Sumit Sharma, Vivek Katiyar

**Affiliations:** 1 General Surgery, Institute of Medical Sciences, Banaras Hindu University (BHU), Varanasi, IND

**Keywords:** blunt trauma abdomen, gastric pneumatosis, gastric ulcer, nonoperative management, portal venous gas

## Abstract

Gastric pneumatosis with portal venous gas (PVG) is an unusual finding after blunt trauma, often regarded as an ominous sign of ischemia or perforation. We report the case of a 54-year-old man who sustained a fall and presented with mild abdominal pain. CT revealed gastric pneumatosis and PVG along with a liver injury, while endoscopy showed a 3 × 3 cm fundic ulcer (Forrest IIc). He remained hemodynamically stable, without peritonitis or biochemical evidence of ischemia, and was managed nonoperatively with proton pump inhibitors, antibiotics, and supportive care. He recovered uneventfully, with complete resolution of imaging findings and ulcer healing on follow-up endoscopy. This case highlights that, although rare, trauma-associated gastric pneumatosis with PVG does not invariably mandate surgery, and nonoperative management can be successful in carefully selected, stable patients under close monitoring.

## Introduction

Gastric pneumatosis (air within the gastric wall) and portal venous gas (PVG) are rare but clinically significant radiologic findings. Gastric pneumatosis may arise from benign causes such as mucosal injury, elevated intraluminal pressure, or instrumentation, while it may also indicate more severe conditions like emphysematous gastritis or ischemia requiring urgent surgical intervention [1]. PVG, historically regarded as a harbinger of necrotic bowel, has also been documented in nonischemic and benign conditions [2,3].

The coexistence of gastric pneumatosis and PVG following blunt abdominal trauma is exceedingly rare, with only a handful of cases reported in the literature. In such settings, these findings have traditionally prompted surgical exploration due to concerns for hollow viscus perforation or ischemia [4]. However, emerging evidence suggests that in carefully selected trauma patients who remain hemodynamically stable and lack peritoneal signs, nonoperative management can be a safe and effective option [5].

Here, we present a rare case involving blunt abdominal trauma resulting in gastric pneumatosis, PVG, and a 3 × 3 cm fundal gastric ulcer (Forrest IIc), managed successfully without surgery. This case underscores the importance of individualized patient assessment rather than reflexive operative decisions in the context of alarming imaging findings.

## Case presentation

A 54-year-old man presented to the emergency department after a fall from a height of approximately 15 feet. The patient complained of left upper limb pain and mild abdominal discomfort. He denied vomiting, hematemesis, or melena. There was no history of previous gastrointestinal bleeding, peptic ulcer disease, or endoscopic interventions.

On examination, the patient was alert and oriented. Vitals on arrival were blood pressure 138/76 mmHg, heart rate 102 beats/min, respiratory rate 18/min, oxygen saturation 98% on room air, and afebrile. The abdomen was mildly distended, with generalized tenderness and guarding, but without rigidity or rebound tenderness. Bowel sounds were present. There were no signs of peritonitis. Examination of other systems revealed tenderness over the left forearm and chest wall without deformity.

Laboratory investigations at presentation are charted in Table 1.

**Table 1:** Laboratory findings at presentation AST (SGOT): aspartate aminotransferase (serum glutamic oxaloacetic transaminase); ALT (SGPT): alanine aminotransferase (serum glutamic pyruvic transaminase); g/dL: grams per deciliter; mg/dL: milligrams per deciliter; U/L: units per liter; mmol/L: millimoles per liter; cells/mm³: cells per cubic millimeter.

Test	Result	Units	Reference range
Hemoglobin	9.5	g/dL	13.5-17.5 (adult male)
Total leukocyte count	6,340	cells/mm³	4,000-10,000
Platelet count	122	×10^⁹^/L	150-400
Serum creatinine	2.03	mg/dL	0.7-1.3
Serum lactate	1.4	mmol/L	0.5-2.0
AST (SGOT)	82	U/L	0-40
ALT (SGPT)	23	U/L	0-40
Total bilirubin	1.2	mg/dL	0.2-1.2
Direct bilirubin	0.7	mg/dL	0.0-0.3
Alkaline phosphatase	40	U/L	40-129
Amylase	526	U/L	30-110
Lipase	28	U/L	13-60

**Figure 1:**
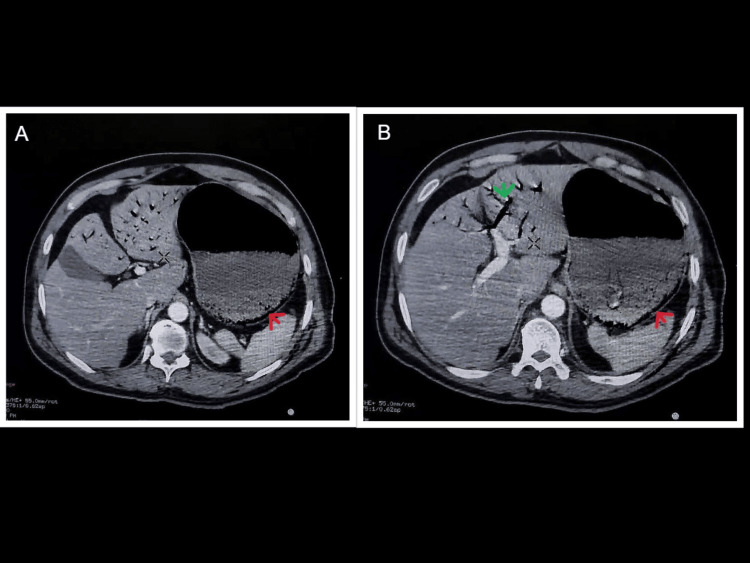
Axial section of CECT whole abdomen A. Gastric pneumatosis marked with a red arrow. B. Portal venous gas marked with a green arrow and gastric pneumatosis with a red arrow. CECT, contrast-enhanced computed tomography.

**Figure 2:**
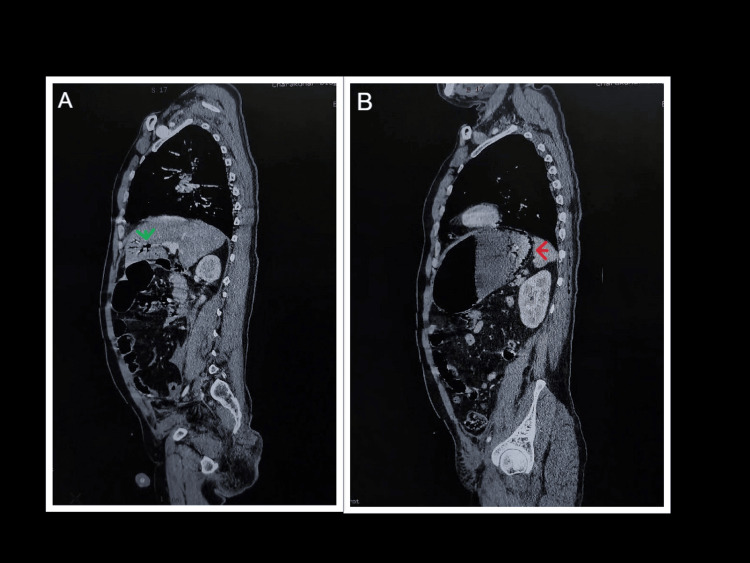
Sagittal section of CECT whole abdomen A. Portal venous gas marked with a green arrow. B. Gastric pneumatosis marked with a red arrow. CECT, contrast-enhanced computed tomography.

Contrast-enhanced computed tomography (CECT) of the thorax and abdomen revealed a Grade I liver injury with a subcapsular hematoma. A fracture of the left ninth rib was noted. There was evidence of gastric pneumatosis with air within the wall of the greater curvature and fundus of the stomach, as well as in the left portal venous branches. No free intraperitoneal air or fluid collection was identified (Figures 1, 2).

**Figure 3:**
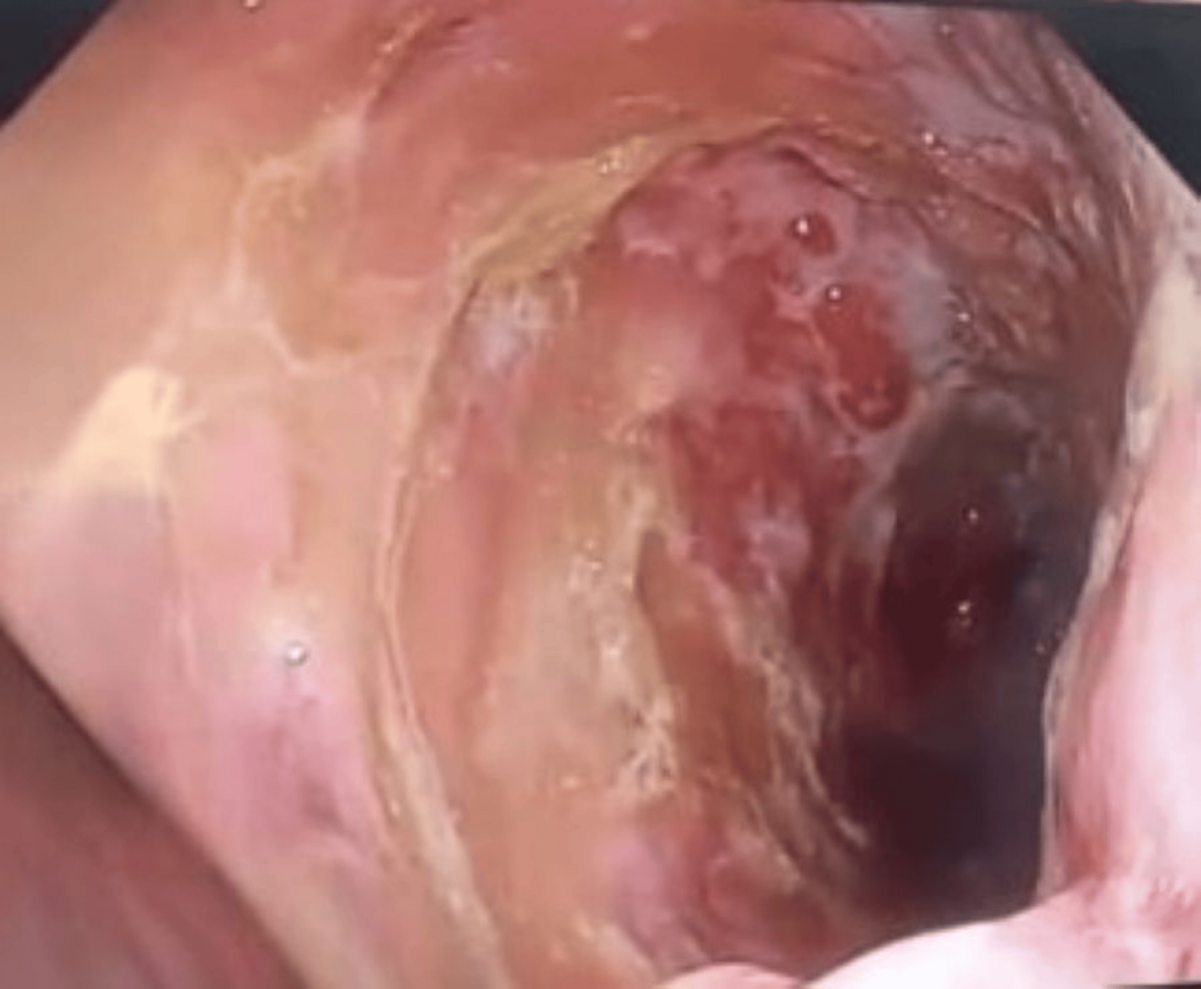
Endoscopic image showing a large excavated ulcer covered with fibrinous exudates and surrounded by highly erythematous mucosa and erosions (Forrest Type IIc)

Upper gastrointestinal endoscopy performed on Day 2 revealed a 3 × 3 cm ulcer in the fundus of the stomach, with a clean base (Forrest IIc), and no active bleeding. No perforation or necrosis was visualized (Figure 3).

Management was nonoperative, consisting of nil per oral (NPO) status for 48 hours, nasogastric decompression, intravenous fluids, high-dose proton-pump inhibitor (PPI) infusion, and broad-spectrum intravenous antibiotics. Analgesia was provided, and serial abdominal examinations were performed. Laboratory parameters and vitals were monitored closely. Oral liquids were initiated on Day 3, advancing to a soft diet by Day 4 as symptoms improved. Serial abdominal examinations remained unremarkable. The patient was discharged on Day 6 in stable condition with advice for outpatient follow-up. At the two-week review, he was asymptomatic and tolerating a normal diet.

## Discussion

Gastric pneumatosis with PVG is an unusual finding in trauma and has traditionally been regarded as an ominous sign of ischemia or perforation requiring urgent surgery [1-4]. However, recent reports indicate that these radiological findings are not always synonymous with irreversible pathology, and in selected patients, can be managed successfully without surgery [5-9].

Only a few cases managed nonoperatively have been described to date, highlighting the uniqueness of our case. Table 2 shows that trauma-associated gastric pneumatosis with PVG has been managed nonoperatively when patients were hemodynamically stable, without peritoneal signs, and had no imaging evidence of transmural ischemia. Management typically involved bowel rest, nasogastric decompression, high-dose PPIs, antibiotics, and close monitoring, with favorable outcomes in all such cases. Conversely, surgical cases (Table 3) usually involved unstable vitals, metabolic derangement, peritonitis, or radiological evidence of necrosis, prompting resection of the affected segment [10,11].

**Table 2:** Reported cases of gastric pneumatosis with portal venous gas managed nonoperatively HPVG: hepato-portal venous gas; NPO: nil per oral; NG: nasogastric.

Reference	Patient details	Clinical findings at presentation	Vitals at presentation	Imaging/Findings	Management	Outcome/Key point
Sen et al., 2013 (India) [5]	29-year-old male, high-speed motorbike crash	Mild upper abdominal pain, no peritonitis	Stable	Gastric intramural air, mucosal disruption, HPVG	NG decompression, IV antibiotics, analgesics	Uneventful recovery without surgery
Stuijvenberg et al., 2009 (Netherlands) [6]	Adult, blunt abdominal trauma	Abdominal discomfort, no tenderness or guarding	Stable	Gastric pneumatosis, intestinal pneumatosis, HPVG	Observation, serial CT	No complications; managed without surgery
Barai et al., 2016 (India) [7]	18-year-old male, fall from height	Epigastric pain, no peritonitis	Stable	Gastric emphysema, Grade II liver injury	NPO, NG suction, IV fluids, antibiotics	Discharged stable, benign course
Trondsen et al., 2009 (Norway) [8]	Adult, blunt trauma	Abdominal pain, mild distension	Stable	Gastric pneumatosis, HPVG	Conservative treatment	No deterioration; safe nonoperative course

**Table 3:** Reported cases of gastric pneumatosis with portal venous gas managed surgically HPVG: hepato-portal venous gas.

Reference	Patient details/Context	Clinical findings at presentation	Vitals at presentation	Imaging/Findings	Management	Outcome/Notes
Petrovic I, et al., 2021 (Croatia) [10]	73-year-old man, acute epigastric pain and hematemesis	Tender epigastrium, mild guarding	BP 110/70, HR 96, afebrile	Gas in gastric wall, HPVG, gastric wall necrosis	Partial gastrectomy + Roux-en-Y esophagojejunostomy	Pathology: emphysematous gastritis with necrosis; recovered well
Dibra R et al., 2020 (Italy) [11]	60-year-old woman	Abdominal tenderness, absent bowel sounds	Hypotensive, tachycardic	Intestinal pneumatosis, HPVG	Exploratory laparotomy with resection of ischemic small bowel	Required bowel resection; emphasizes emergent surgical role

The difference between the two groups underscores that the decision to operate should be guided more by physiological status and clinical findings than by imaging alone. Our patient’s stable vitals, absence of peritonitis, normal lactate, and CT showing gastric wall air with PVG but no free intraperitoneal air matched the low-risk profile seen in conservatively managed cases. PVG results from gas entering the portal venous system through mesenteric or gastric veins. In this case, a large fundal ulcer likely caused mucosal disruption, and the trauma-induced transient intragastric pressure surge could have facilitated gas entry into the wall and portal circulation without causing transmural perforation. The absence of systemic toxicity or ischemic changes explains the benign course [12-14].

Three factors favored nonoperative management: stable hemodynamics without shock or severe sepsis, no biochemical or clinical evidence of transmural ischemia, and contained pathology confirmed on endoscopy (Forrest IIc ulcer, no active bleed or perforation). While laboratory investigations showed an elevated amylase of 526 U/L and mild renal impairment with a serum creatinine of 2.03 mg/dL, these were not indicative of severe pathology. The normal lipase level of 28 U/L and CT imaging effectively ruled out clinically significant pancreatic trauma, and mild renal impairment was managed adequately with supportive intravenous fluids. Mild elevation in direct bilirubin was considered insignificant and a transient reflection of the concurrent mild liver injury on the initial CT imaging. Close clinical observation, serial examinations, and timely supportive measures allowed early detection of any deterioration and full recovery without operative intervention, while supportive measures (PPI infusion, antibiotics, bowel rest) promoted mucosal healing and resolution of pneumatosis. This aligns with modern literature advocating for individualized decision making in PVG and gastric pneumatosis, rather than a blanket mandate for surgery [2,3,11].

## Conclusions

Gastric pneumatosis with PVG is an uncommon finding after blunt abdominal trauma and often raises concern for serious intra-abdominal pathology requiring urgent surgery. However, as demonstrated in this case, when the patient is hemodynamically stable and lacks peritoneal signs, and imaging does not show evidence of transmural ischemia or perforation, nonoperative management can be a safe and effective alternative. Careful patient selection, close clinical monitoring, and timely supportive interventions are critical for successful outcomes. This report adds to the growing body of evidence that not all cases of gastric pneumatosis with PVG mandate operative intervention, highlighting the importance of individualized decision-making.

## References

[1] Liebman PR, Patten MT, Manny J, Benfield JR, Hechtman HB (1978). Hepatic-portal venous gas in adults: Etiology, pathophysiology and clinical significance. Ann Surg.

[2] Abboud B, El Hachem J, Yazbeck T, Doumit C (2009). Hepatic portal venous gas: Physiopathology, etiology, prognosis and treatment. World J Gastroenterol.

[3] Nelson AL, Millington TM, Sahani D (2009). Hepatic portal venous gas: The ABCs of management. Arch Surg.

[4] Kingsley DD, Albrecht RM, Vogt DM (2000). Gastric pneumatosis and hepatoportal venous gas in blunt trauma: Clinical significance in a case report. J Trauma.

[5] Sen I, Samarasam I, Chandran S, Mathew G (2013). Gastric intramural and portal venous gas following blunt abdominal injury. Arch Trauma Res.

[6] Stuijvenberg M, van der Meer NJ, Crolla RM (2009). Pneumatosis intestinalis with gastric pneumatosis and hepatoportal venous gas in blunt abdominal trauma: A case report. Eur J Trauma Emerg Surg.

[7] Barai B, Mandal A, Chakroborty P, Sarkar K, Bhattacharyya S, Ali J (2016). Gastric emphysema following blunt trauma abdomen-A rare entity. Sch J Med Case Rep.

[8] Trondsen E, Skattum J, Quigstad E, Meidell N, Naess PA, Gaarder C (2009). Gastric pneumatosis and hepatic portal venous gas after blunt trauma does not mandate surgery. Injury Extra.

[9] Adu-Gyamfi KO, Amakye D, Kudaravalli P, Shahsavari D, Yap JEL (2022). Ischemic gastritis with gastric pneumatosis and portal venous gas. Am J Gastroenterol.

[10] Petrovic I, Sremac M, Grbavac D (2021). Intramural stomach gas with hepatic portal venous gas indicating spontaneous stomach necrosis. Middle East J Dig Dis.

[11] Dibra R, Picciariello A, Trigiante G (2020). Pneumatosis intestinalis and hepatic portal venous gas: Watch and wait or emergency surgery? A case report and literature review. Am J Case Rep.

[12] Bak MA, Rajagopalan A, Ooi G, Sritharan M (2023). Conservative management of emphysematous gastritis with gastric mucosal ischaemia: A case report. Cureus.

[13] Beger B, Kızılyıldız BS, Ozdemir O (2021). Hepatic portal venous gas after blunt abdominal trauma in a child. East J Med.

[14] Iwamuro M, Takenaka R, Toyokawa T (2024). Endoscopic and clinical features of gastric emphysema. Sci Rep.

